# Supplementary material for: In Vivo Gastroprotective and Antidepressant Effects of Iridoids, Verbascoside and Tenuifloroside from Castilleja tenuiflora Benth
Source: Molecules. 2019 Apr 2;24(7):1292. doi: 10.3390/molecules24071292 (PMC6479932; doi:10.3390/molecules24071292)
Supplement: Supplementary file 1 [file molecules-24-01292-s001.pdf]

Supplementary Material

In Vivo Gastroprotective and Antidepressant Effects of Iridoids, Verbascoside and Tenuifloroside from *Castilleja tenuiflora* Benth

Ricardo López-Rodríguez [1,2], Maribel Herrera-Ruiz [2], Gabriela Trejo-Tapia [1,*], Blanca Eda Domínguez-Mendoza [3], Manases González-Cortazar [2] and Alejandro Zamilpa [2,*]

[1] Centro de Desarrollo de Productos Bióticos, Instituto Politécnico Nacional, Col. San Isidro, Yautepec, Morelos C.P. 62731, Mexico; richard_lorr@hotmail.com

[2] Centro de Investigación Biomédica del Sur, Instituto Mexicano del Seguro Social, Argentina No. 1, Col. Centro, Xochitepec, Morelos C.P. 62790, Mexico; cibis_herj@yahoo.com.mx (M.H.-R.); gmanases2000@gmail.com (M.G.-C.)

[3] Centro de Investigaciones Químicas, Universidad Autónoma del Estado de Morelos, Av. Universidad 1001, Col. Chamilpa, Cuernavaca, Morelos C.P. 62209, Mexico; bed@uaem.mx

* Correspondence: gttapia@ipn.m (G.T.-T.); azamilpa_2000@yahoo.com.mx (A.Z.); Tel.: +52-735-394-2020 (G.T.-T.); Tel.: +52-777-361-21-55 (A.Z.)

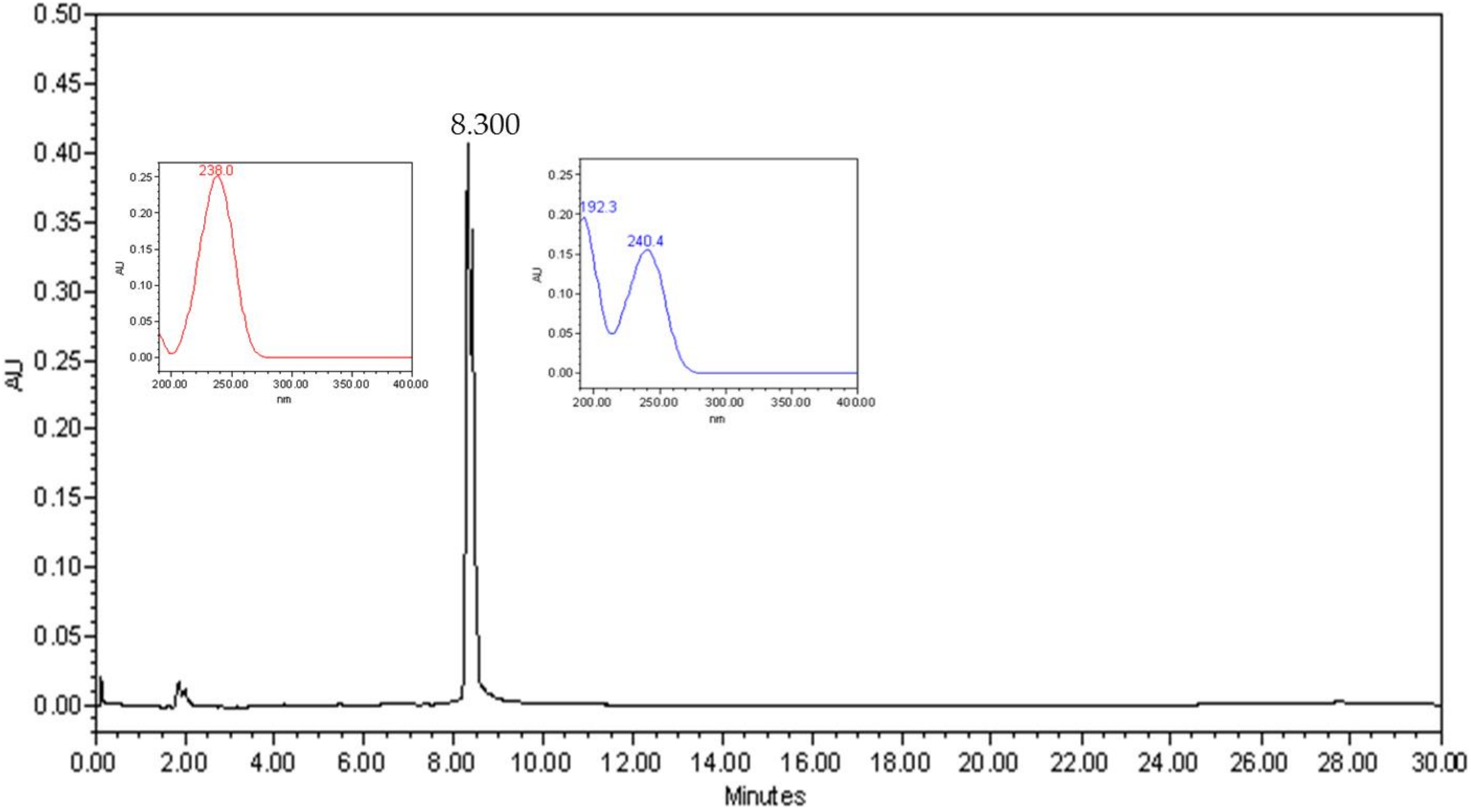

Figure S1. HPLC chromatogram and UV light spectrum of iridoids (**GP/MS**).

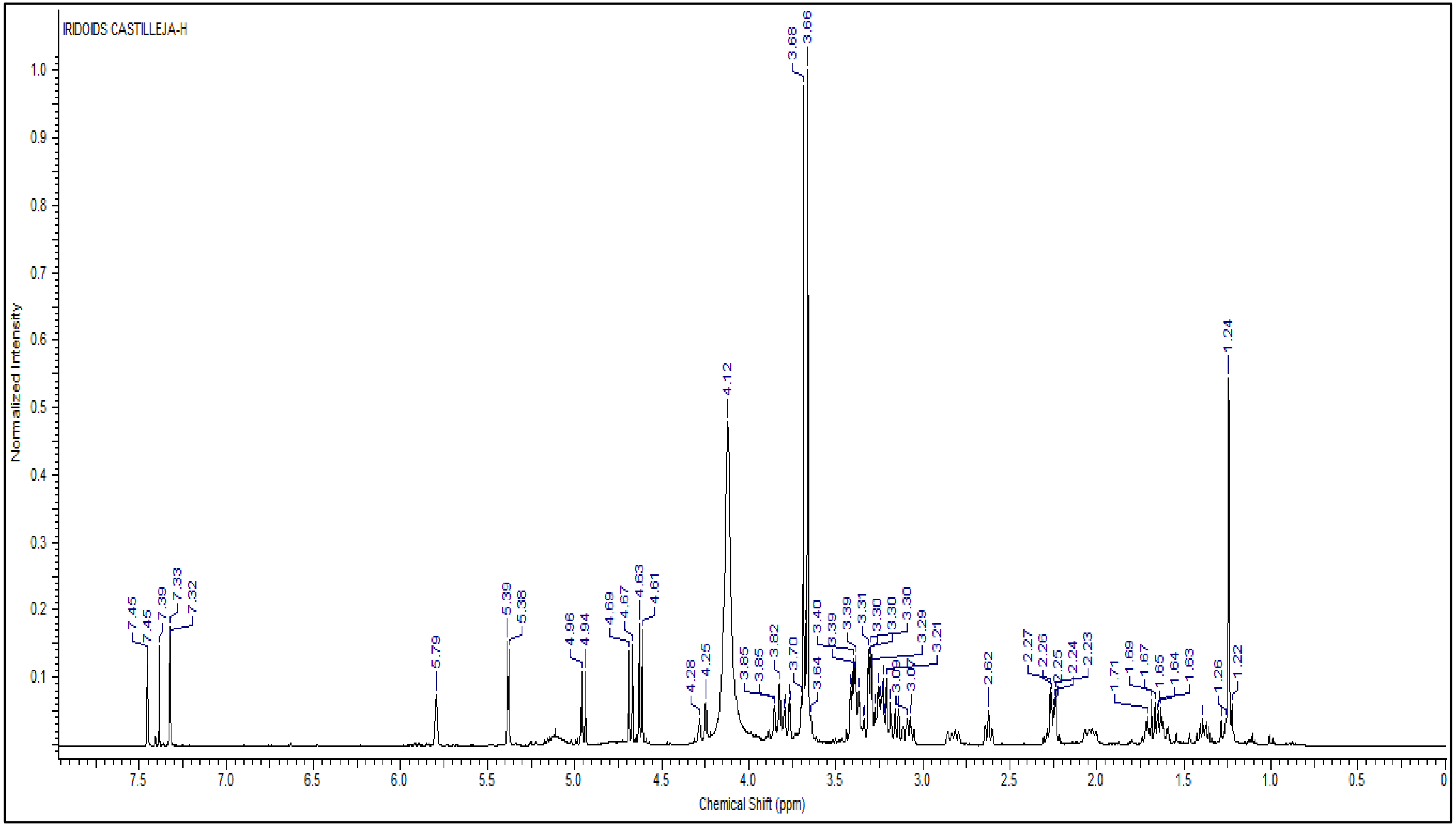

Figure S2. ^{1}H-NMR (DMSO, 400 MHz) Iridoids (**GP/MS**).

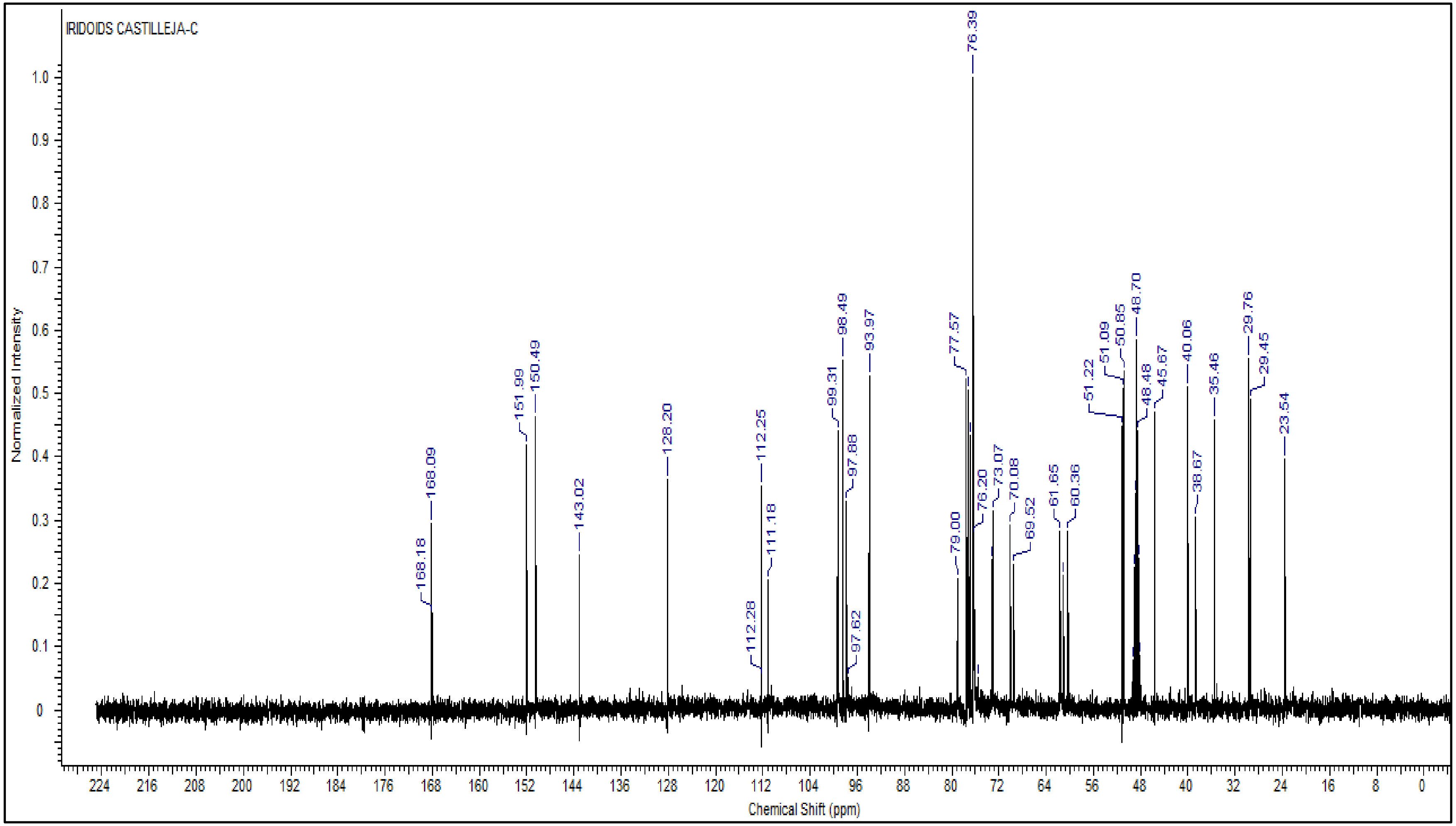

Figure S3. ^{13}C-NMR (DMSO, 100 MHz) Iridoids (**GP/MS**).

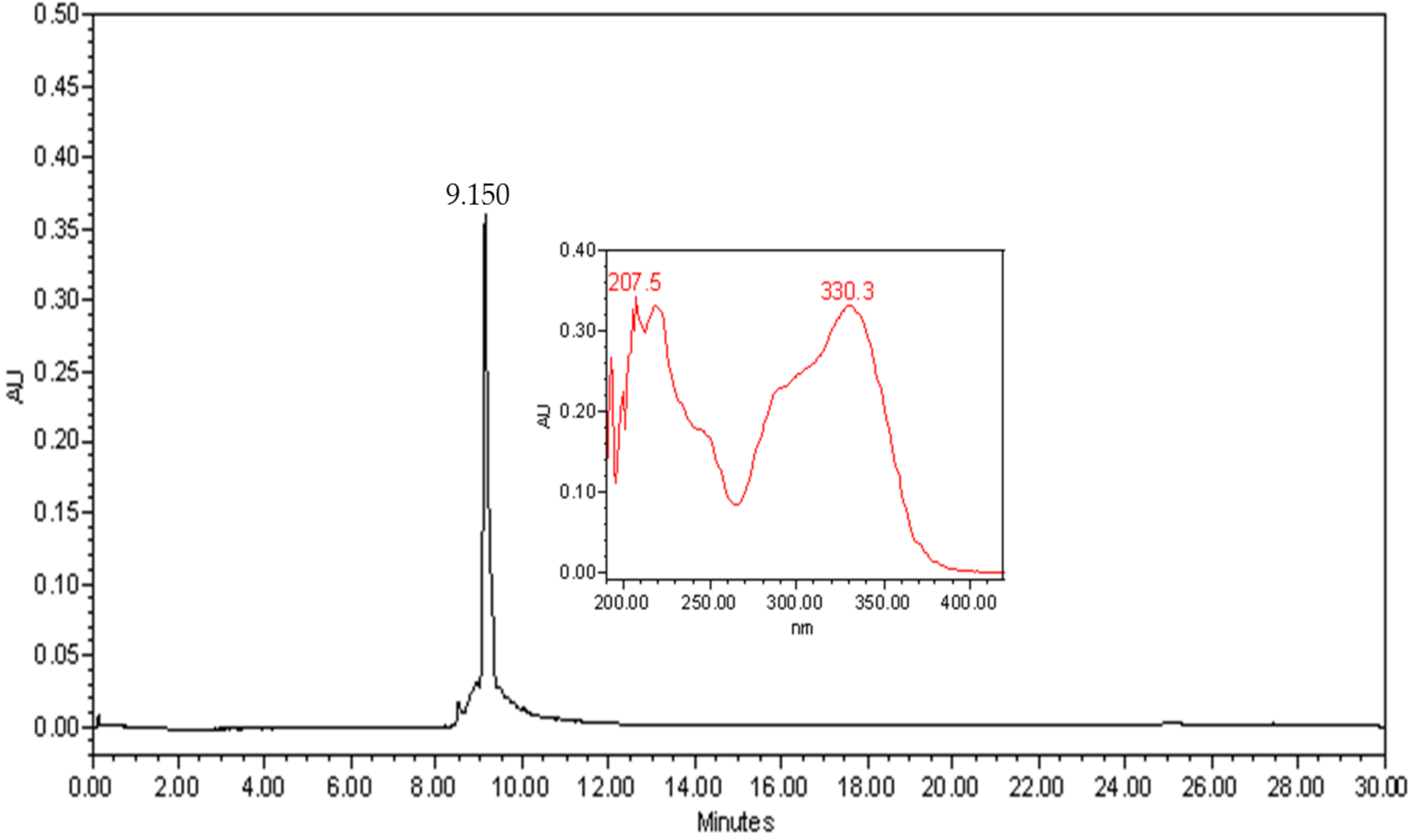

Figure S4. HPLC chromatogram and UV light spectrum of verbascoside (**VB**).

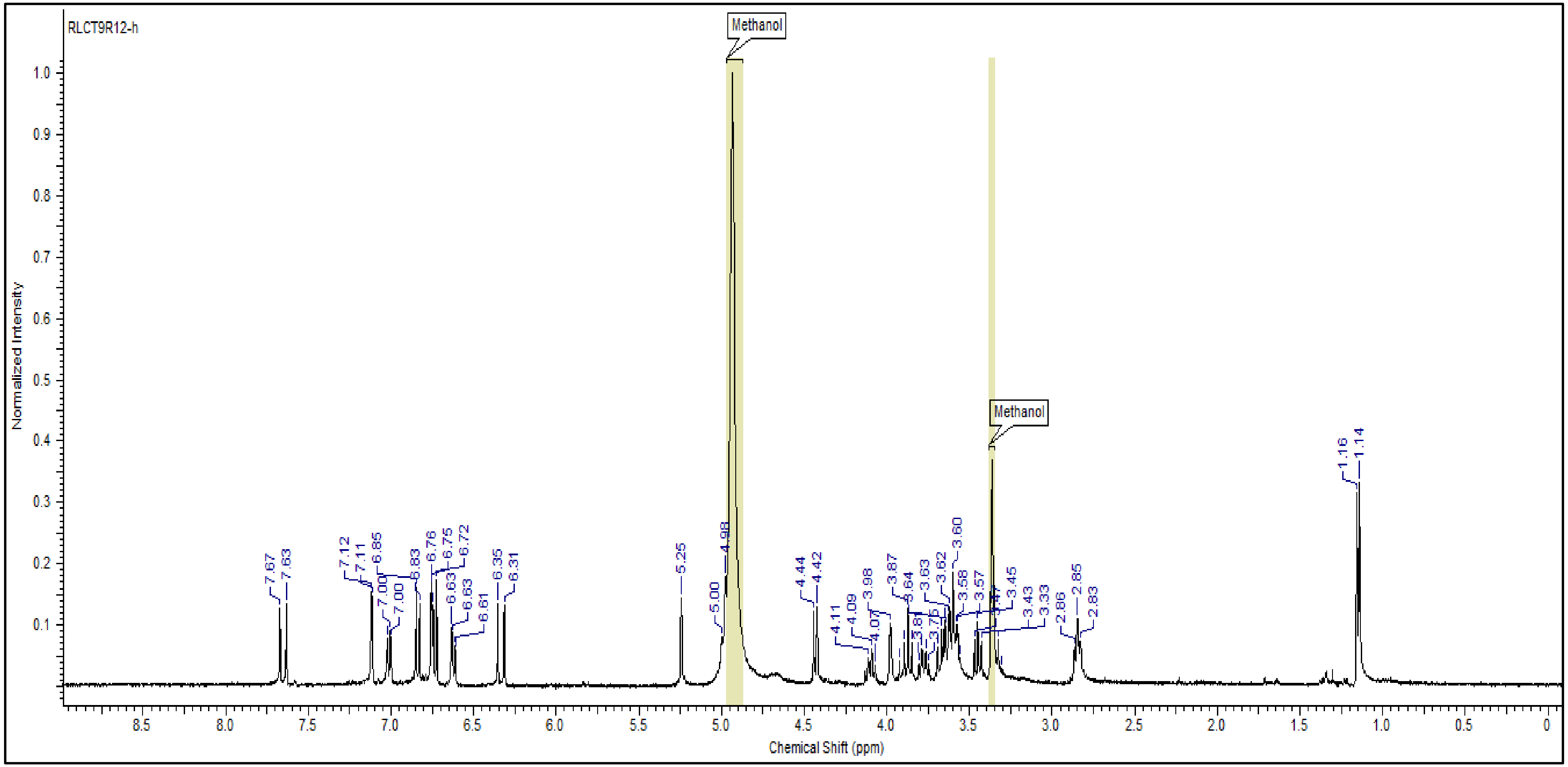

Figure S5. ^{1}H-NMR (CD_3OD, 400 MHz) Compound (**VB**).

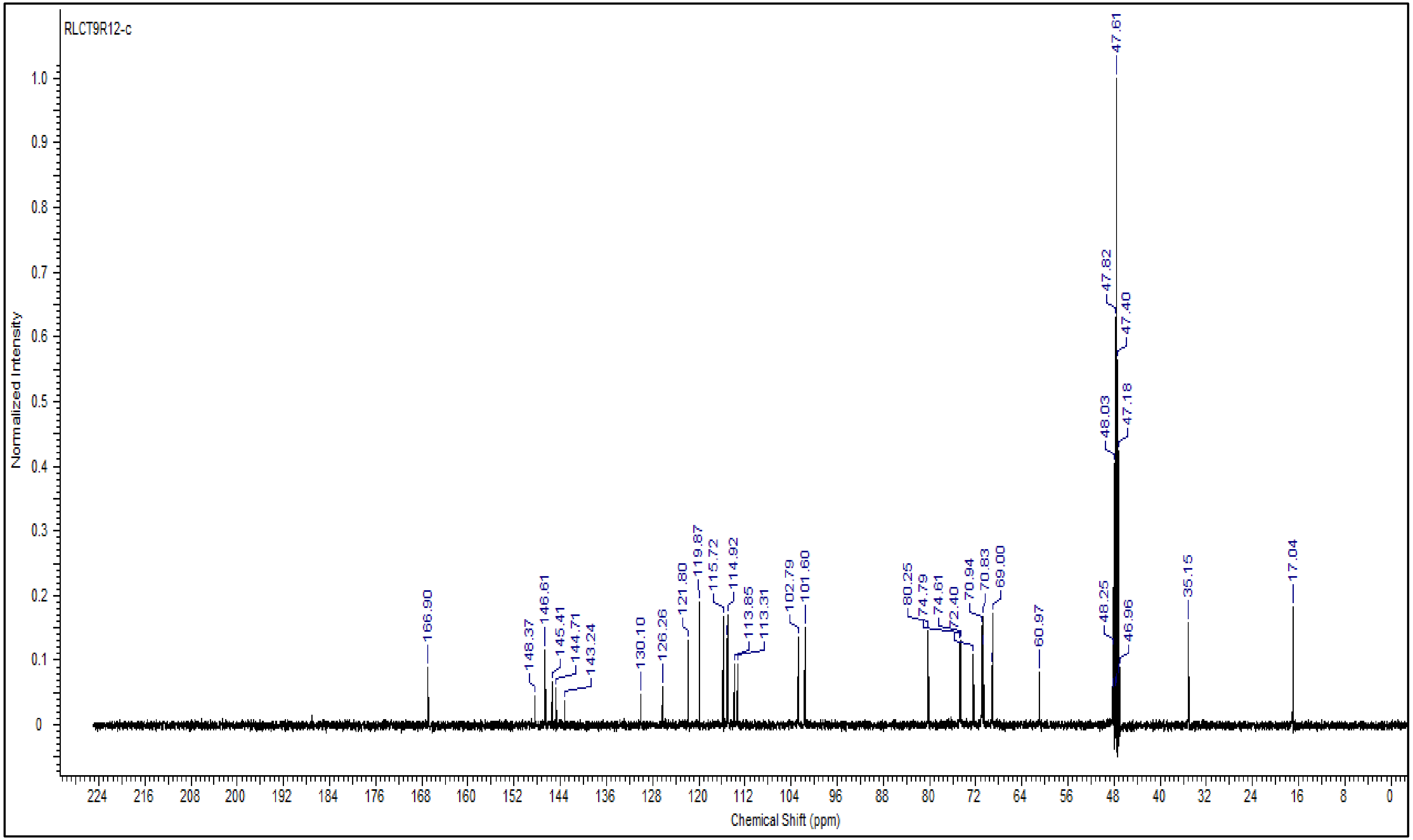

Figure S6. ^{13}C-NMR (CD_3OD, 100 MHz) Compound (**VB**).

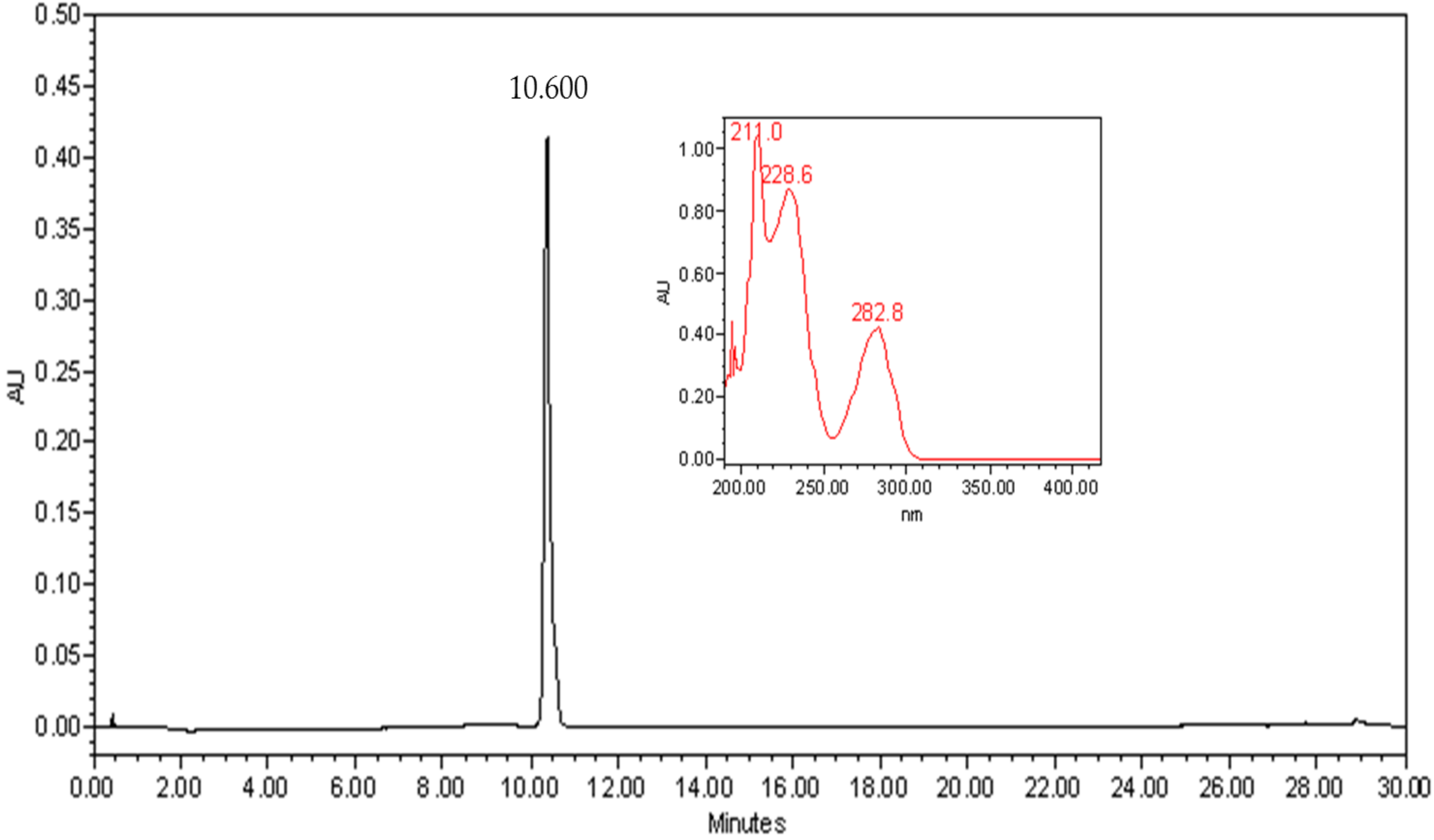

Figure S7. HPLC chromatogram and UV light spectrum of tenuifloroside (**TN**).

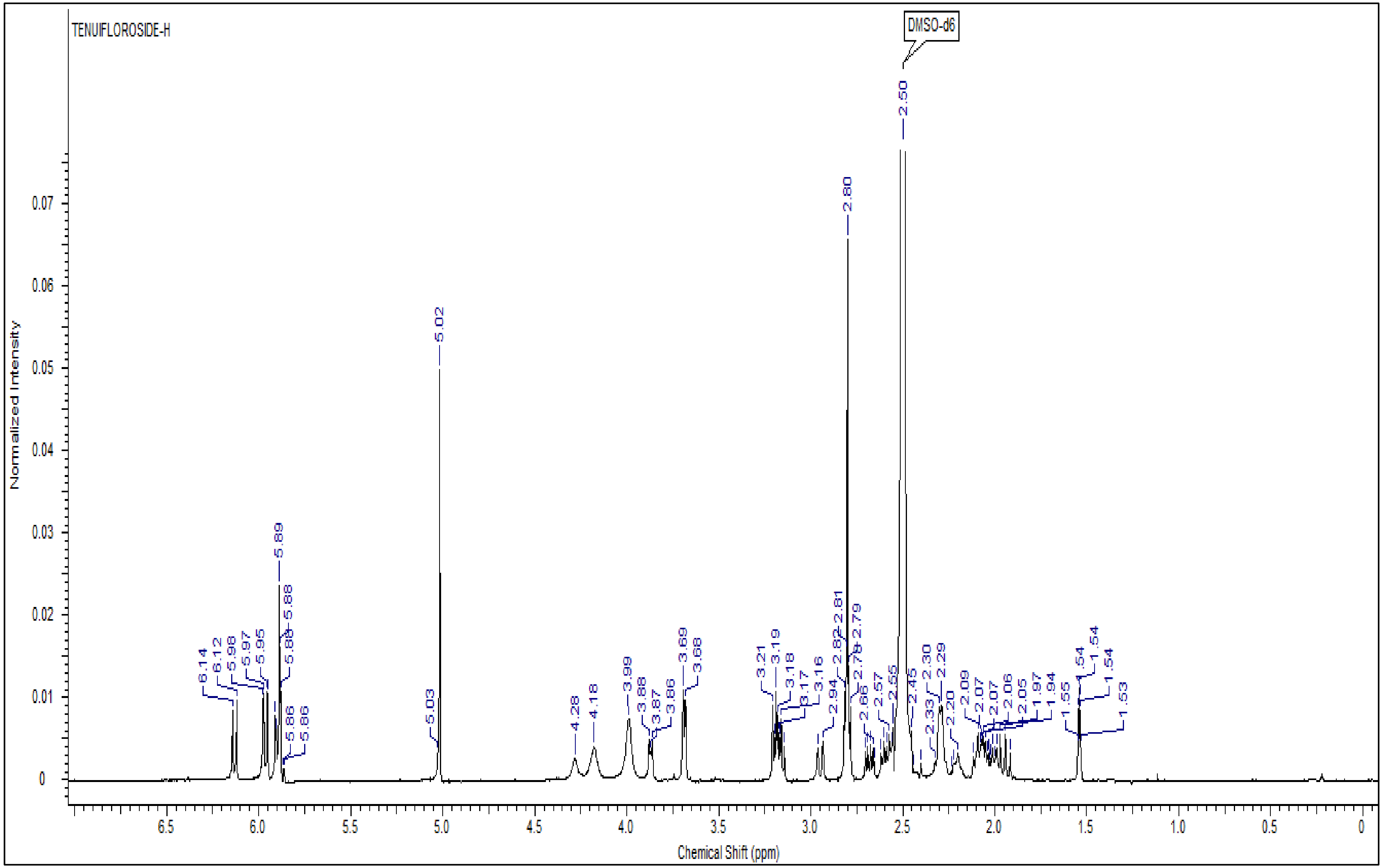

Figure S8. ^{1}H-NMR (DMSO, 400 MHz) Compound (**TN**).

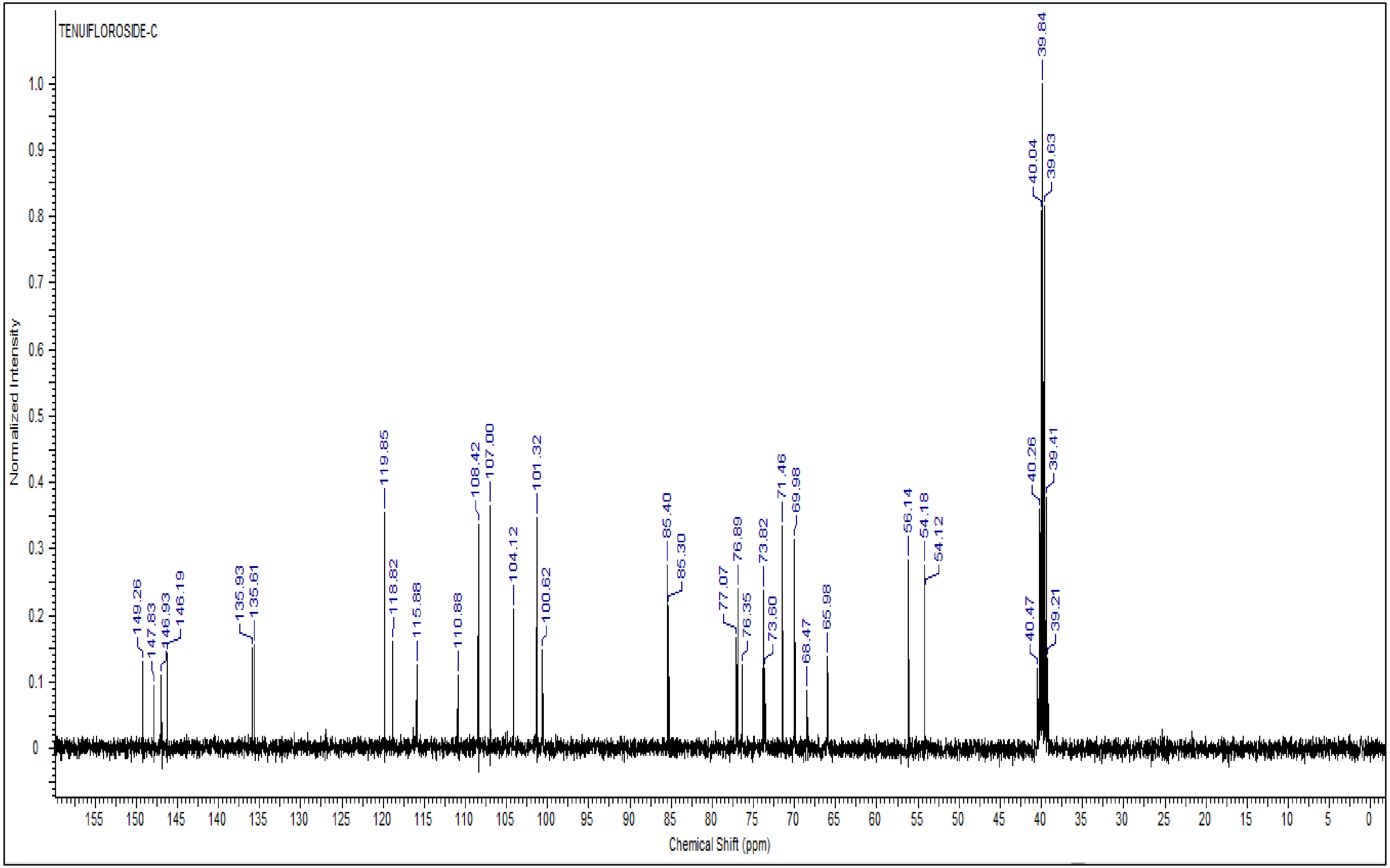

Figure S9. ^{13}C-NMR (DMSO, 100 MHz) Compound (**TN**).

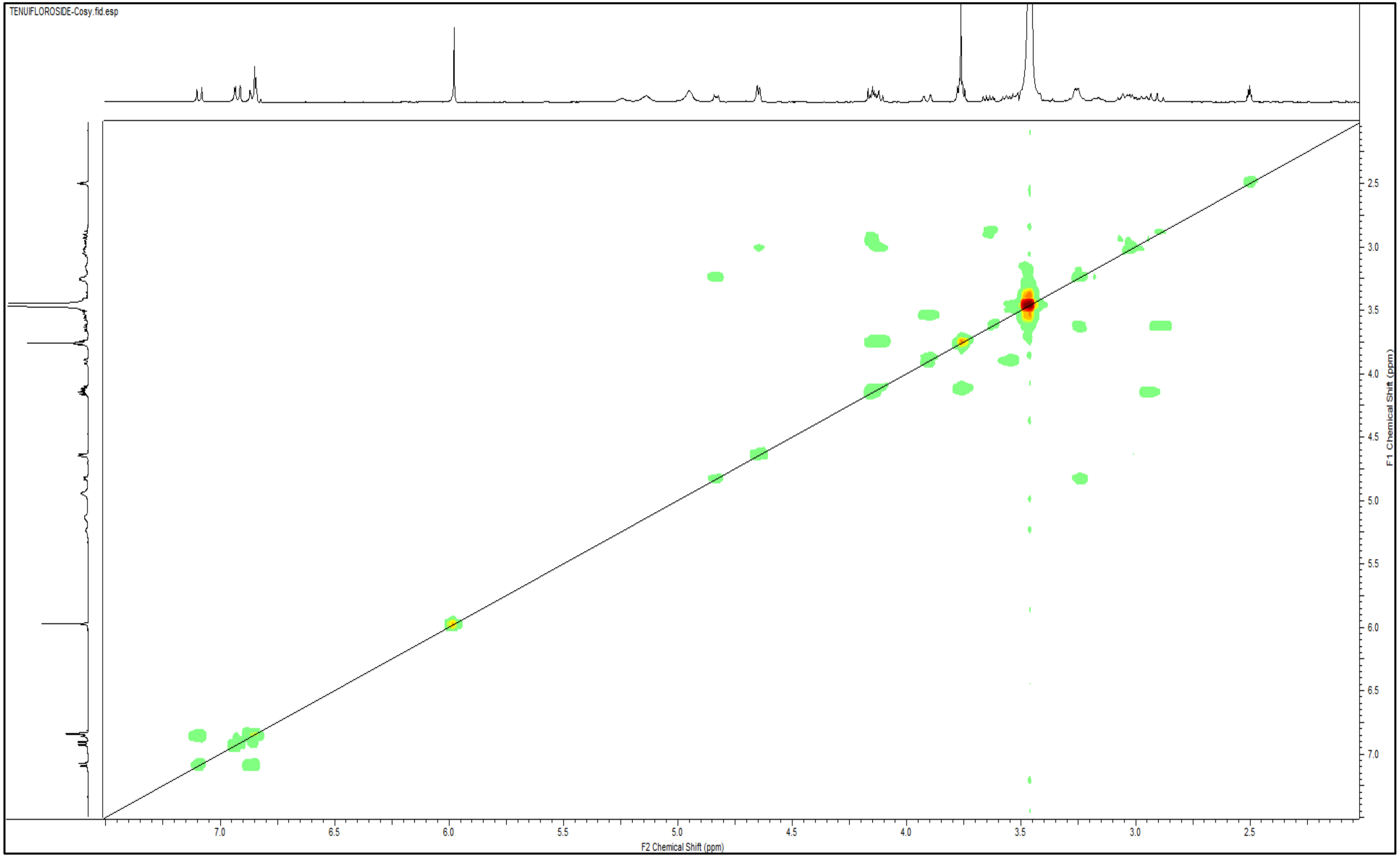

Figure S10. ^{1}H-^{1}H (COSY) NMR (DMSO, 400 MHz) Compound (**TN**).

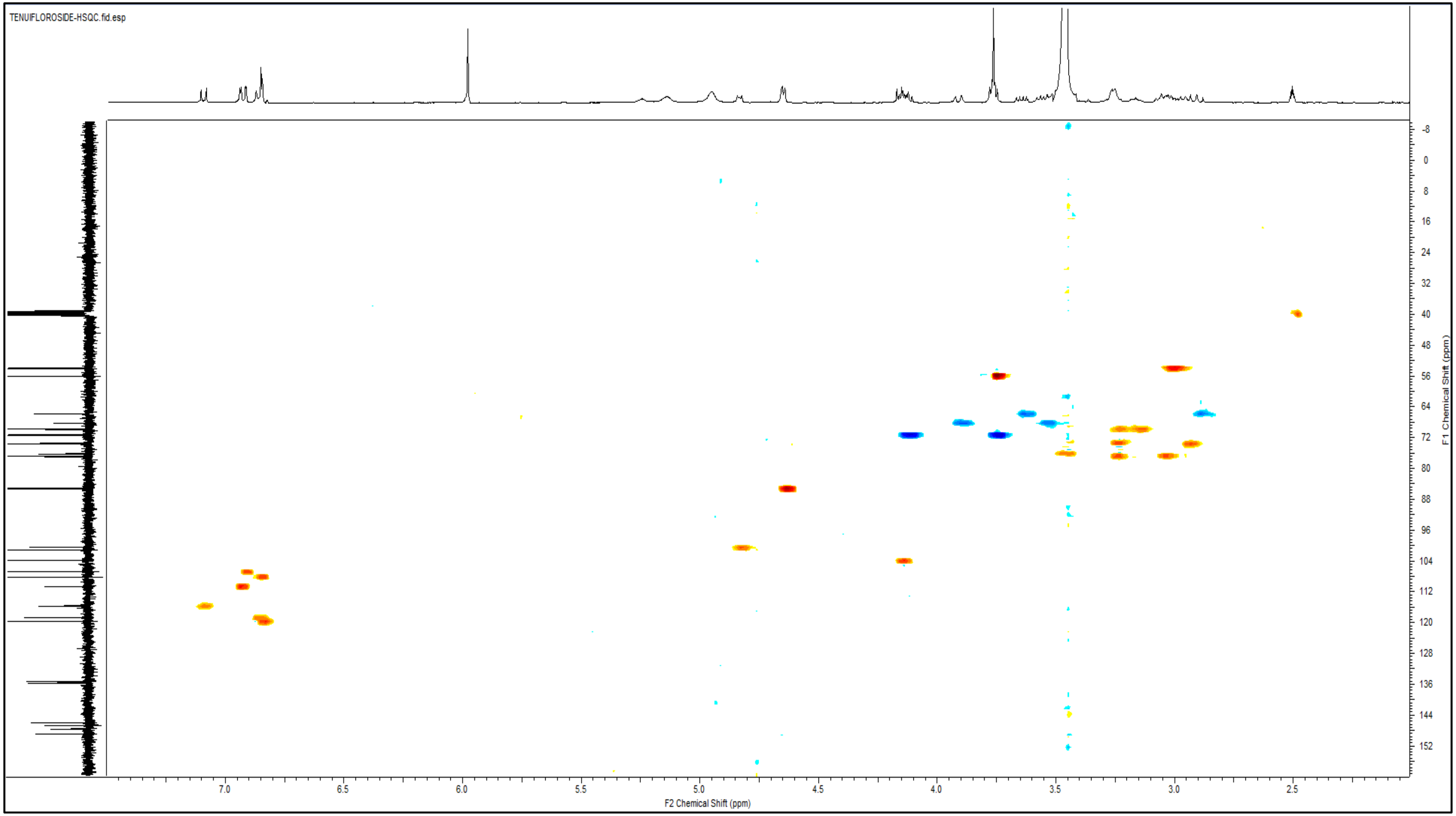

Figure S11. ^{1}H-^{13}C HSQC (DMSO, 400 MHz) Compound (**TN**).

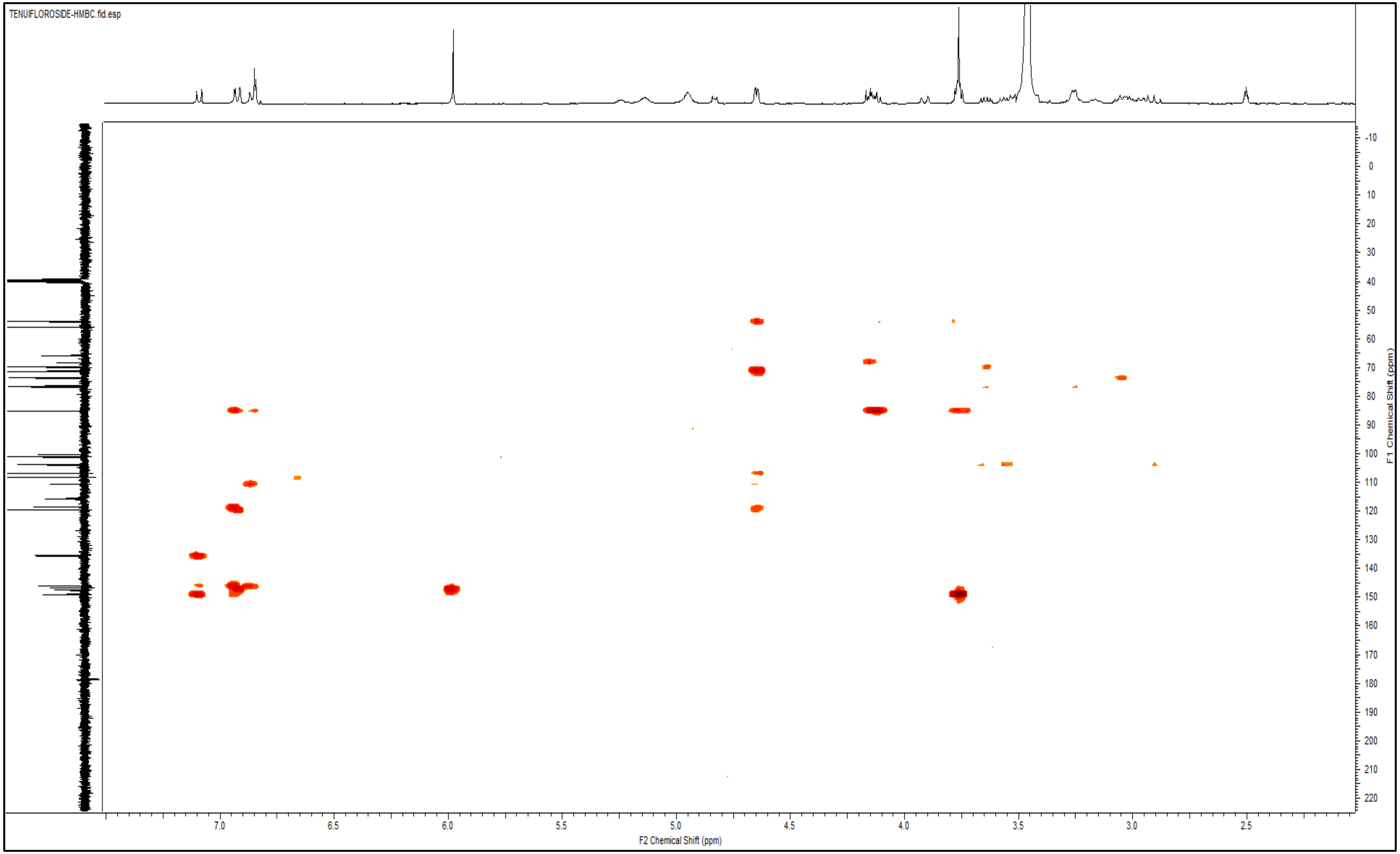

Figure S12. HMBC (DMSO, 400 MHz) Compound (**TN**).
